# Identifying genes preferentially expressed in undifferentiated embryonic stem cells

**DOI:** 10.1186/1471-2121-8-37

**Published:** 2007-08-28

**Authors:** Xiajun Li, Philip Leder

**Affiliations:** 1 Department of Genetics, Harvard Medical School, Boston, MA 02115, USA

## Abstract

**Background:**

The mechanism involved in the maintenance and differentiation of embryonic stem (ES) cells is incompletely understood.

**Results:**

To address this issue, we have developed a retroviral gene trap vector that can target genes expressed in undifferentiated ES cells. This gene trap vector harbors both GFP and Neo reporter genes. G-418 drug resistance was used to select ES clones in which the vector was integrated into transcriptionally active loci. This was then followed by GFP FACS profiling to identify ES clones with reduced GFP fluorescence and, hence, reduced transcriptional activity when ES cells differentiate. Reduced expression of the GFP reporter in six of three hundred ES clones in our pilot screening was confirmed to be down-regulated by Northern blot analysis during ES cell differentiation. These six ES clones represent four different genes. Among the six integration sites, one was at *Zfp-57 *whose gene product is known to be enriched in undifferentiated ES cells. Three were located in an intron of a novel isoform of *CSL/RBP-Jkappa *which encodes the key transcription factor of the LIN-12/Notch pathway. Another was inside a gene that may encode noncoding RNA transcripts. The last integration event occurred at a locus that may harbor a novel gene.

**Conclusion:**

Taken together, we demonstrate the use of a novel retroviral gene trap vector in identifying genes preferentially expressed in undifferentiated ES cells.

## Background

Stem cells offer hope of potential therapies for diseases as disparate as diabetes, Parkinson's and Alzheimer's disease. Naturally, knowledge of the intrinsic properties of stem cells must be gained before the hope of using stem cells for therapeutic purposes becomes a reality. In particular it will be important to know how these cells that retain the ability to continuously self-renew in a multipotential state can be maintained in this undifferentiated state.

There are more than a dozen different kinds of stem cells in mammals, including those that retain the ability of totipotential differentiation, the so-called embryonic stem cells or pluripotential stem cells [1]. Recent data suggest that some adult stem cells are more plastic than originally thought [1]. Not only can certain types of adult stem cells be induced to differentiate along several cell lineages (termed transdifferentiation), it has also been observed that differentiated cells can be reverted to stem cell-like cells (termed dedifferentiation) and redirected to other cell lineages [2]. Although it has been recently shown that cell fusion could provide an alternative explanation for the phenomenon of transdifferentiation [3,4], it is still consistent with the notion that there are some key regulators in stem cells that keep them in an undifferentiated pluripotent state.

We are interested in identifying these determinants since they are likely to play an important role in the maintenance, proliferation, survival or differentiation of stem cells. ES cells should express the determinants typical of all stem cells. They can be easily maintained in culture and are amenable to both genetic and molecular manipulations. Thus, we have chosen to use mouse ES cells as a model system in which to isolate determinants that maintain stem cells in their undifferentiated state or promote the differentiation of stem cells into a certain lineage. One assumption for such stem cell determinants is that at least some are only expressed in undifferentiated stem cells, but not in differentiated cells. Indeed, the *Oct-4 *and the *Nanog *genes, both of which encode a homeodomain transcription factor, are required for the establishment of the undifferentiated state of ES cells and are rapidly down-regulated in differentiated cells [5-10]. The expression of another transcription factor, *Rex-1*, also correlates with the undifferentiated state of ES cells [11,12]. To identify additional genes that behave similarly to *Oct-4*, *Nanog *and *Rex-1*, we have used a gene trap approach coupled with GFP FACS analysis to screen for genes that are expressed in undifferentiated ES cells, but dramatically down-regulated in the differentiated cells. Among these genes, presumably some play an essential role as determinants of the undifferentiated state or factors in promoting the differentiation of ES cells into a particular lineage.

Gene trap is a genetic approach that utilizes promoter-less reporters to target endogenous functional genes [13-16]. It could be either plasmid- or retrovirus-based. The advantage of using a retroviral vector is that retroviral infection normally results in a single integration event. We have constructed a novel promoter-less retroviral gene trap vector harboring both GFP and Neo reporter genes that can only be expressed when the vector is integrated into an active endogenous gene. By utilizing this vector, coupled with GFP FACS profiling of 300 ES clones, we have successfully isolated 30 candidate clones that displayed less GFP fluorescence when the ES cells differentiated. We confirmed by RT-PCR or Northern blot analysis that the genes trapped in six of the candidate ES clones were down-regulated during ES cell differentiation.

The ease of using the gene trap approach and GFP FACS profiling, plus the simplicity of identifying the trapped genes, make this vector a suitable choice to target and isolate ES cell-specific genes in mice. Similar to previous studies [17-22], it can be applied to other cell types to identify developmentally regulated genes or genes that are responsive to certain growth factors.

## Results

### Construction of a retroviral gene trap vector that allows GFP FACS profiling as well as G-418 drug selection

**Figure 1:**
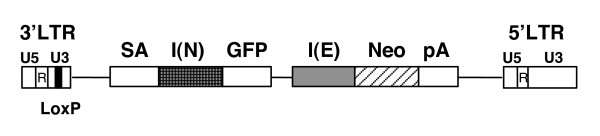
**Diagram of the gene trap vector eGeoN/E+pA**. Neo, Neo resistance gene; GFP, cDNA encoding green fluorescent protein; pA, polyA addition signal derived from the gene of bovine growth hormone (bGH); SA, splice acceptor sequence present in intron 2/exon 3 of *Bcl-2*; I(E), IRES sequence from encephalomyocarditis virus (EMCV); I(N), IRES sequence from the 5' UTR region of the *NF-kappaB *repressing factor (*NRF*) gene; 3'LTR and 5'LTR, long terminal repeat regions of the RET vector modified from murine Moloney leukemia virus [23]. Both 3'LTR and 5'LTR are comprised of U5, R and U3. The regulatory sequence in the U3 region of 3'LTR was deleted and replaced with a loxP site which could be recognized by Cre recombinase.

As a first step toward the goal of isolating ES cell-specific genes, we designed a novel murine Moloney leukemia virus (MMLV)-based retroviral gene trap vector. This vector harbors a strong splice acceptor (SA) site followed by GFP and Neo reporter genes (Figure 1). The SA site is derived from the intron 2/exon 3 region of *Bcl-2 *[23]. Each reporter is under the control of an independent internal ribosome entry site (IRES) (Figure 1). The regulatory sequence in the U3 region of the 3'LTR was deleted and replaced with a LoxP site which is duplicated upon retrovirus-mediated integration. Most of the gene trap vector can be removed by Cre recombinase [23]. Upon reverse transcription and retroviral integration, the U3 region of the 3'LTR is duplicated. Neither the 3'LTR nor the 5'LTR of the integrated gene trap vector contained any viral enhancer or promoter. Therefore, neither reporter is expressed unless the vector is integrated into a transcriptionally active endogenous gene. Due to the presence of the SA site, reporters are co-transcribed as a single fusion transcript after being directly spliced to an exon of a transcribed endogenous gene that lies 5' to the inserted vector. The GFP and Neo reporters are translated as two independent protein products since the IRES sequence placed immediately in front of each reporter gene can initiate translation independent of the 5' upstream sequences of the fusion transcript. Accordingly, the infected cells and their progeny carrying this integrated vector display green fluorescence as well as G-418 drug resistance. G-418 drug resistance conferred by the expression of the Neo reporter gene can be used to select for ES clones in which a transcriptionally active endogenous gene has been targeted. GFP fluorescence resultant from expression of the GFP reporter gene allows us to screen for ES clones that display significant down-regulation of green fluorescence upon ES cell differentiation.

### Isolation of ES clones in which the trapped genes are down-regulated during ES cell differentiation

**Figure 2:**
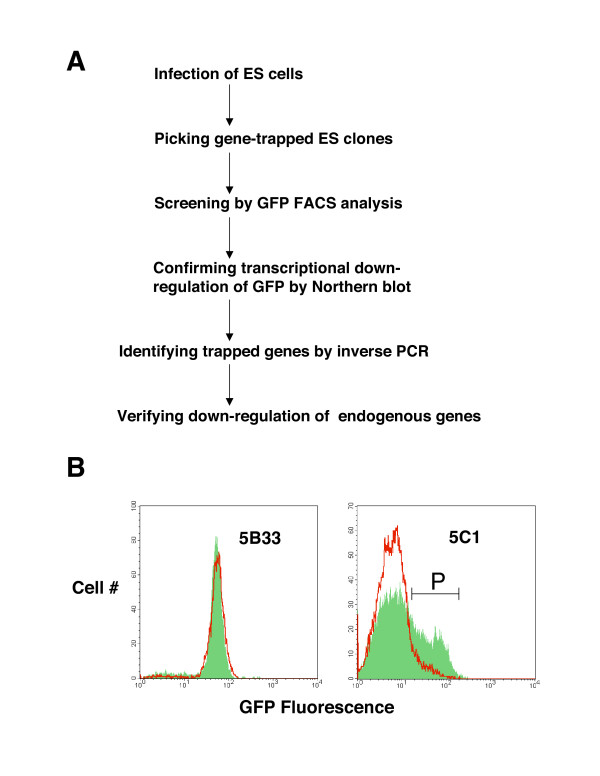
**G-418 drug resistance and GFP fluorescence were used to isolate the trapped ES clones whose trapped genes are down-regulated during ES cell differentiation**. (A) Flow chart for the experimental procedure.(B) GFP FACS profiling of the ES clones. FACS plots of the ES clone 5B33 (left panel) and ES clone 5C1 (right panel) are shown with both the undifferentiated populations of cells (the green filled histograms) and differentiated populations of cells (the red unfilled histograms). Undifferentiated ES cells were grown in the presence of LIF and feeder fibroblast cells and the differentiated cells were grown without LIF and feeder fibroblast cells. "P" marks the GFP-positive population of ES cells present in the undifferentiated cells of the ES clone 5C1.

The schematic procedure used to isolate candidate ES clones is illustrated in Figure 2A. Viral supernatant of this gene trap vector was harvested from phoenix packaging cells [24] and directly applied onto TC1 mouse ES cells grown on a layer of feeder fibroblast cells. After selection with 260 μg/ml of G-418 for 10 days, approximately 10–20 resistant colonies were obtained from a 10-cm dish. These clones were picked individually and transferred into a 24-well plate seeded with feeder fibroblast cells. In total we picked about 300 independent ES clones in a pilot screening experiment.

If the endogenous gene is turned off when ES cells differentiate, the expression of GFP and Neo reporters in the trapped locus will not persist and these cells will lose both green fluorescence and drug resistance. We have taken advantage of one of the conspicuous traits of ES cells; they have the tendency to spontaneously differentiate. In the presence of feeder fibroblast cells and leukemia inhibitory factor (LIF), a significant fraction of ES cells remain undifferentiated. By contrast, almost all ES cells become differentiated within four days without feeder fibroblast cells and LIF. Two populations of cells were derived for each ES clone: undifferentiated cells grown in the presence of feeder fibroblast cells and LIF or differentiated cells grown without either feeder fibroblast cells or LIF. These two populations of cells were compared for each ES clone for the level of GFP fluorescence by FACS profiling.

About three hundred ES clones were screened to identify those that displayed significant reduction in GFP fluorescence when differentiated. About 40% of the G-418-resistant ES clones we examined did not show any fluorescence in FACS analysis. We reasoned that enzyme-based drug selection is more sensitive than GFP fluorescence and thus not all G-418-resistant ES clones displayed GFP fluorescence. For the rest of the ES clones, expression of the GFP reporter appeared to remain unchanged in most ES clones when comparing undifferentiated cells and their differentiated counterparts. For example, in the ES clone 5B33 very little difference in GFP fluorescence was detected between the undifferentiated and differentiated populations of cells (Figure 2B, left panel). Nevertheless, about 30 out of 300 ES clones screened exhibited significant down-regulation of GFP expression by FACS analysis. The highly GFP-positive portion of the cells from the undifferentiated sample of the ES clone 5C1 was almost completely missing in the differentiated sample (Figure 2B, right panel).

### Confirming the down-regulation of the candidate ES clones at the transcript level by Northern blot

**Figure 3:**
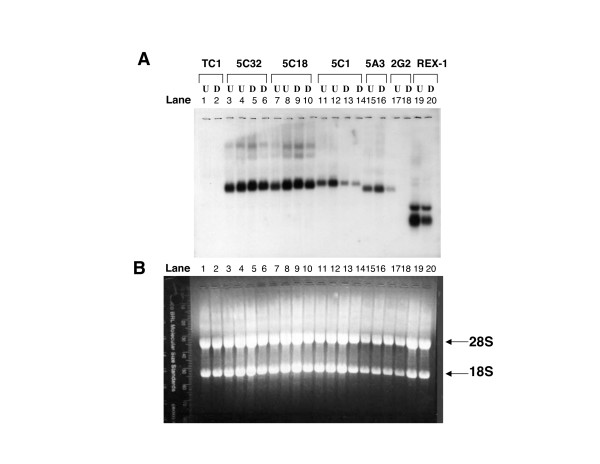
**Northern blot analysis of the candidate ES clones**. (A) The exposed X-ray film of the Northern blot with a GFP cDNA fragment as the probe. U, Undifferentiated ES cell population. D, Differentiated ES cell population. TC1, wild-type ES cells. REX-1, an ES clone with the REX1::EGFP reporter transgene. 5C32, 5C18, 5C1, 5A3 and 2G2 are gene-trapped ES clones isolated in this study. (B) The RNA-agarose gel under an ultraviolet (UV) lamp. Approximately 10 μg of total RNA was loaded for the U and D samples of the control samples and each ES clone, except that 5 μg of total RNA was applied for the ES clone 5A3 and 2G2. The positions of 18S and 28S ribosomal RNAs on the agrose gel are marked.

**Figure 5:**
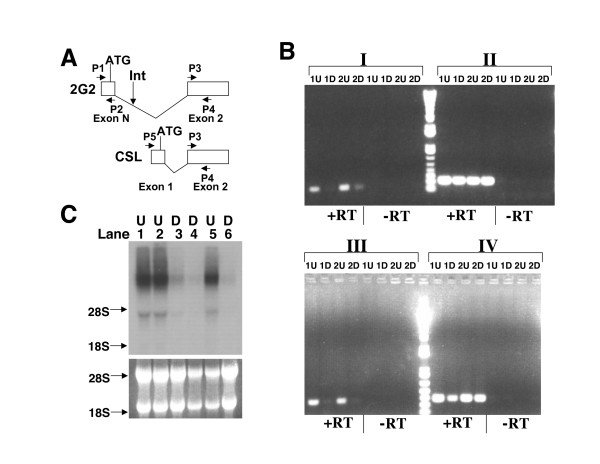
**Verifying the down-regulation of the endogenous genes during ES cell differentiation**. (A) Diagrams are shown for the 5' portion of the novel embryonic isoform (upper, 2G2) as well as the most common isoform (lower, CSL) of *CSL/RBP-Jkappa*. The first exon (Exon N) of the novel embryonic isoform is located upstream of the second exon (Exon 2) which is common to all known isoforms. The predicted initiation codon "ATG" is indicated above the first exons and the primers (P1, P2, P3, P4 and P5) used in the semi-qauntitative PCR analysis below (Figure 5B) are also shown. "Int" stands for the retroviral integration site in the ES clone 2G2, which is located within the first intron of the novel embryonic isoform. Retroviral integration occurred in the same intron for two other ES clone 5C25 and 6B13. (B) Verification of the reduced expression of the novel early embryonic isoform of *CSL/RBP-Jkappa *in the differentiated cells (D) versus undifferentiated (U) cells by quantitative RT-PCR analysis. 1U and 2U, two independent total RNA samples from the undifferentiated wild-type ES cells. 1D and 2D, two independent total RNA samples from the differentiated cells. Equal amounts of total RNA was applied to every PCR reaction in each primer set. Four different primer sets (I, II, III, IV) were used in this quantitative PCR analysis. I, both primers (P1 and P2) are derived from the first exon unique to this novel isoform (Figure 5A). II, both primers (P3 and P4) are common to all the isoforms of *CSL/RBP-Jkappa*. III, the forward primer (P1) is derived from the first exon and the reverse primer (P4) is complementary in sequence to the second exon, which is common to all isoforms of *CSL/RBP-Jkappa*. IV, the forward primer (P5) corresponds to the first exon of the most common isoform of *CSL/RBP-Jkappa *and the reverse primer (P4) is complementary in sequence to the common second exon. "+RT" indicates that reverse transcriptase (RT) was included in the first-strand cDNA synthesis. "-RT" indicates negative controls in which no reverse transcriptase was added in the first-strand cDNA synthesis. (C) Confirming the down-regulation of the novel gene trapped in the ES clone 5C11 by Northern blot with a gene-specific probe. Total RNA samples were derived from three independent populations (Lanes 1, 2, 5) of the undifferentiated (U) wild-type ES cells and three independent populations (Lanes 3, 4, 6) of the differentiated (D) wild-type ES cells. The gel positions of 18S and 28S ribosomal RNA transcripts are marked with arrows.

The intensity of GFP fluorescence of every ES clone as well as the degree of down-regulation in GFP FACS profiles upon ES cell differentiation varied greatly among these candidate clones. To confirm that down-regulation of GFP fluorescence reflects decreased transcription of the GFP reporter gene, we resorted to Northern blot analysis to examine these candidate ES clones directly at the transcript level with a GFP cDNA fragment as the probe (Figure 3 and Supplemental Figure S1, see Additional file 1). Except for the ES clone 5A3, 1B6 and 6A29, the rest of 22 ES clones shown on Northern blotting displayed significant reduction of GFP fluorescence in FACS profiling as exemplified by the ES clone 5C1 (Figure 2B) and 5C32 (Supplemental Figure S2, see Additional file 1). We used an ES clone harboring a REX-1::EGFP transgene as the positive control in which the expression of the GFP reporter gene is under the control of *Rex-1 *regulatory sequences [25,26]. We found that this ES clone underwent the expected reduction of GFP fluorescence during ES cell differentiation based on GFP FACS analysis (data not shown). Indeed, the GFP transcript expressed in these ES clones was also reduced in the differentiated cells versus the undifferentiated cells (compare Lane 20 with Lane 19 in Figure 3). Similar to the ES clone transfected with the REX-1::EGFP reporter construct, the amount of fusion transcript appeared to be down-regulated in the trapped ES clone 5C1 and 2G2 as shown in Figure 3 (compare Lanes 13, 14 with Lanes 11, 12 and Lane 18 with Lane 17). In fact, transcription of the gene trapped in the ES clone 2G2 appeared to be completely shut off when ES cells differentiated. Overall, six ES clones out of 30 candidate ES clones were confirmed to display down-regulation of the reporter genes at the transcript level (Figure 3 and Supplemental Figure S1, see Additional file 1) and they are illustrated in Table 1.

**Table 1:** Illustration of the trapped genes proven to be significantly down-regulated in the differentiated ES cells versus undifferentiated ES cells

Trapped ES clone	Matched Gene	EST Origin
2G2, 5C25, 6B13	A novel *CSL *isoform	Eight-cell embryo, Germ cell, ES cell
5C1	*Zfp-57*	Embryo, Germ cell, ES cell
5C11	Novel	Blastocyst, ES cell
4B1	Unknown	

### Determining the molecular identities of genes trapped in the candidate ES clones

To determine the molecular identities of the trapped genes, we used an inverse PCR approach to isolate the retroviral integration site of these candidate ES clones together with the flanking genomic sequence (data not shown). This genomic sequence was used to search the available genome databases. Indeed, we identified the integration site for 15 out of 19 candidate ES clones shown in Figure 3 and Supplemental Figure S1 (see Additional file 1) which displayed significant down-regulation of GFP fluorescence by FACS analysis when differentiated and they are illustrated in Table 1 and Table 2. Out of the six ES clones that displayed significant down-regulation of the reporter expression in both GFP FACS profiling and Northern blotting, we were able to match all the integration sites to the mouse genome sequence and predict the correspondingly trapped genes in five of the six ES clones (Table 1).

**Table 2:** Illustration of ES clones that display significant down-regulation in FACS profiling but could not be confirmed by Northern blot

Trapped ES clone	Matched gene (or genomic region)
5C32, 5C18	M96
5B1, 5B5, 5B20, 5A8	CTBP2
2F1 (2 integrations)	UBA52 and LYSMD1
4A8	LYSMD1
5B39	Near CXXC6

One clone resulted from integration in the gene *Zfp-57*. This gene was previously isolated in a screen for genes that were expressed in undifferentiated teratocarcinoma F9 cells but down-regulated upon treatment of F9 cells with retinoic acid [27]. In agreement with our studies, *Zfp-57 *was also found to be expressed in ES cells and down-regulated when differentiated into neurons [28-30]. It encodes a basic protein with multiple zinc fingers and is predominantly localized to the nucleus [27]. Similar to the findings described in a recent paper [30,31], we also found that ZFP-57 contains a putative KRAB box (Li, Youngson, Zhou, Ito, Ferguson-Smith and Leder, unpublished data).

The integration sites for three independent clones were localized to the same genomic locus. Indeed, the gene trap vector was inserted into the same huge intron but at different locations of a novel isoform of *CSL/RBP-Jkappa *(see Figure 5A below) encoding the key transcription factor of the LIN-12/Notch pathway [32]. This isoform differs from other isoforms by using a transcriptional initiation site considerably upstream of those used by the other isoforms. The integration sites for these three ES clones are located in the first intron of this novel isoform (see Figure 5A below). Based on genomic databases, EST clones matching this isoform were found to be present in eight-cell embryos, embryonic germ cells and embryonic stem cells.

The gene trapped in the fifth ES clone matches two EST clones that are expressed in the blastocyst. Analysis of cDNAs for this gene suggests the presence of multiple alternatively spliced isoforms with the characteristics of noncoding RNA molecules (see below).

The identity of the gene trapped in the last ES clone, which was confirmed to be down-regulated when ES cells differentiate, remains to be determined.

### Confirming the identities of the candidate genes by RT-PCR

**Figure 4:**
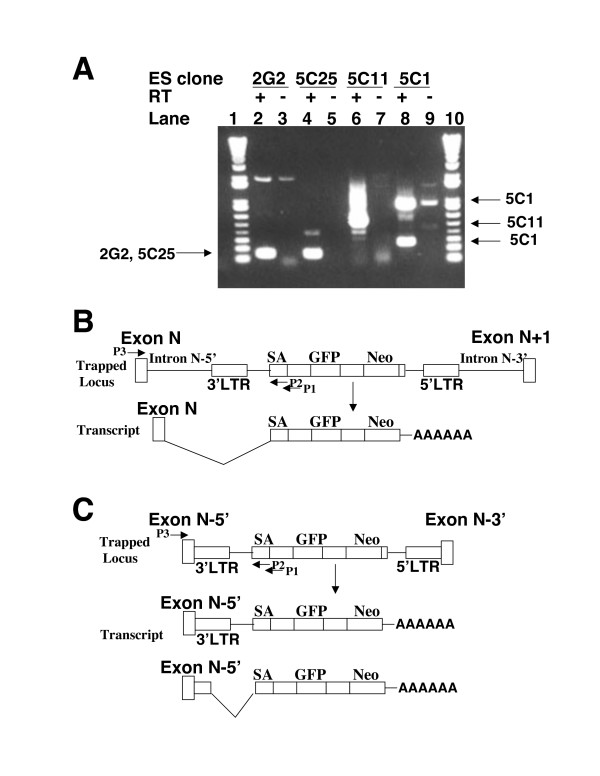
**The identities of the trapped genes were verified by RT-PCR analysis of the fusion transcripts**. (A) RT-PCR products of the four candidate ES clones (2G2, 5C25, 5C11 and 5C1). The primer Xho-Junc (P1), which has a sequence complementary to the junction of the SA site and I(N) sequence in the gene trap vector eGeoN/E+pA, was used for reverse transcription in the first-strand cDNA synthesis. Another primer Bcl-2R2 (P2) complementary to exon 3 of *Bcl-2 *in eGeoN/E+pA was paired with the gene-specific primer (P3) to amplify the cDNAs derived from the fusion transcripts between the endogenous trapped genes and the gene trap vector. The positions of these primers (P1, P2, P3) with respect to the gene trap vector and the trapped endogenous genes are indicated in Figure 4B and Figure 4C. Arrows indicate the agarose gel positions of the expected RT-PCR products. "+", reverse transcriptase (RT) was included in the first-strand cDNA synthesis (Lanes 2, 4, 6, 8). "-", no reverse transcriptase was added to the first-strand cDNA synthesis (Lanes 3, 5, 7, 9). Lane 1 and Lane 11, 1kb plus DNA ladder (Invitrogen/Gibco). (B) Schematic diagrams illustrating the fusion transcripts expressed in the trapped ES clones when the gene trap vector is inserted into an intron of the active endogenous genes (e.g. ES clone 2G2 and 5C25). As shown in the diagrams, the vector is inserted into the Intron N between Exon N and Exon N+1. After the splicing of the fusion transcripts, the splice donor present at the junction of Exon N/Intron N was utilized to join the Exon N directly to the splice acceptor (SA) present in front of the GFP and Neo reporter genes. (C) Schematic diagrams illustrating the fusion transcripts expressed in the trapped ES clones when the gene trap vector is integrated into an exon of the active endogenous genes (e. g. ES clone 5C1, the first exon of *Zfp-57*). As shown in the diagrams, the vector is integrated into Exon N, which results in the split of Exon N into two parts (Exon N-5' and Exon N-3'). A read-through fusion transcript will be generated or the cryptic splice donor site present in the 3'LTR of the vector is utilized to join the Exon N-5' part and portion of the 3'LTR directly to the splice acceptor (SA) present in front of the GFP and Neo reporter genes.

To confirm the identities of the predicted genes trapped in the candidate ES clones, we resorted to RT-PCR analysis of the transcriptional fusion products between the trapped gene and the gene trap vector. This was performed using one primer complementary in sequence to the exon 3 region of *Bcl-2 *found in the gene trap vector and the other one corresponding to the upstream exon of the predicted trapped endogenous gene. In every case, the expected fusion products were obtained for each ES clone from the reverse transcriptase (RT)-treated samples, but not from negative control samples (Figure 4A, compare, for example, Lanes 2 and 3 for the clone 2G2). Thus, the predicted trapped genes were confirmed for these candidate ES clones.

Sequencing of these fusion RT-PCR products not only confirmed our prediction for these trapped genes, but also provided us with the opportunity to observe the exact splicing patterns of fusion transcripts around the integration site. As shown schematically in Figure 4, there are basically three types of transcripts generated depending on whether the integration occurred in an intron or into an exon. When the integration site was localized in an intron of the endogenous gene (Figure 4B), as was the case in the ES clone 2G2 and 5C25, a completely spliced fusion transcript was observed due to the presence of the nearby splice donor sequence at the junction of the exon N and intron N which acted as the donor to the strong splice acceptor site in front of the IRES-EGFP reporter. However, when the gene trap vector was inserted into an exon of the endogenous gene as was observed in the ES clone 5C1 (Figure 4C), two kinds of transcripts were produced: a read-through fusion transcript and a partially spliced fusion transcript. The read-through fusion transcript includes the 5' portion of the interrupted exon (Exon N-5') of the endogenous gene, the 3'LTR region, the SA region and the reporters. The partially spliced fusion transcript was generated when the cryptic splice donor site present in the 3'LTR was utilized to promote splicing of the 5' portion of the interrupted exon (Exon N-5') along with a portion of the 3'LTR in the splice acceptor site in front of the reporters. No matter whether the gene trap vector is inserted into an intron or an exon, fusion transcripts will be generated and both GFP and neo reporter genes will be translated as two independent protein products due to the presence of an IRES sequence in front of each reporter gene.

### Verification of the down-regulation of the endogenous genes

We employed either Northern blot with gene-specific probes or semi-quantitative RT-PCR to test if the expression of the endogenous genes behaves similarly to that of the GFP reporter gene in the correspondingly trapped ES clones.

We utilized a semi-quantitative RT-PCR approach to investigate the novel embryonic isoform of *CSL/RBP-Jkappa *trapped in the ES clone 2G2, 5C25 and 6B13. Four strategies were used: two primers specific to the embryonic isoform (I of Figure 5B), or two primers common to all isoforms (II of Figure 5B), or one primer specific to the embryonic isoform and the other common to all isoforms (III of Figure 5B), or one primer specific to the most common and ubiquitously expressed isoform and the other common to all the isoforms (IV of Figure 5B). In either strategy I or III, when at least one primer is specific to the particular embryonic isoform, a relatively bright band of the expected molecular weight was seen on the agarose gel for the RT-PCR products amplified from two independent total RNA samples (1U and 2U in Figure 5B) of undifferentiated wild-type ES cells. In contrast, only a minimal amount of product was amplified from two corresponding samples obtained from differentiated cells (1D and 2D in Figure 5B). As expected, similar amounts of amplified product were obtained from the corresponding samples when either all the isoforms were amplified (II of Figure 5B) or only the most common one was amplified (IV of Figure 5B). Thus, it appears that this novel embryonic isoform of *CSL/RBP-Jkappa *is preferentially expressed in the undifferentiated ES cells.

Transcriptional down-regulation of the novel gene trapped in the ES clone 5C11 was verified by Northern blot using a gene-specific probe (Figure 5C). Three independent total RNA samples were derived from both the undifferentiated (Lanes 1, 2 and 5 of Figure 5C) as well as the differentiated (Lanes 3, 4 and 6 of Figure 5C) wild-type ES cells. Clearly, many more transcripts were present in all the samples prepared from the undifferentiated ES cells than those from the differentiated cells.

In agreement with published findings [27,28,30], we also confirmed the down-regulation of *Zfp-57 *trapped in the ES clone 5C1 by Northern blot when ES cells differentiate (data not shown).

### The novel gene trapped in the ES clone 5C11 may be a non-coding RNA gene with a very restricted expression pattern

**Figure 6:**
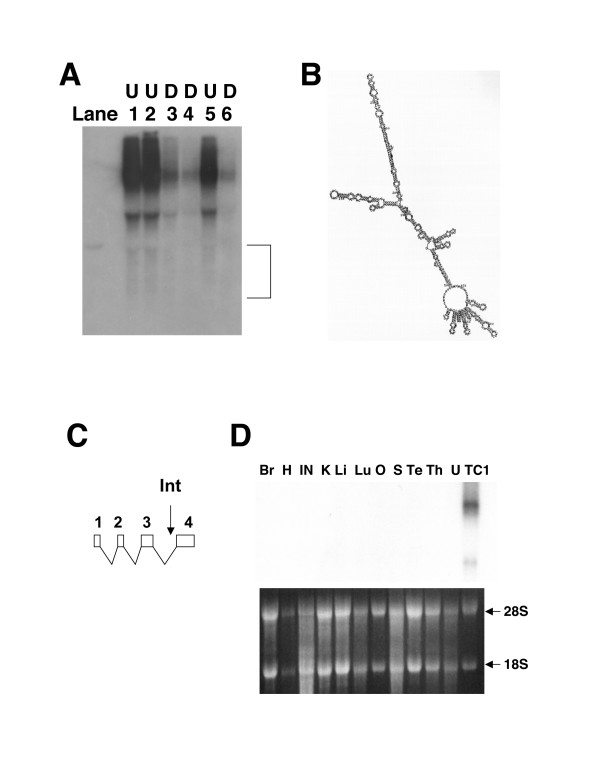
**The novel gene trapped in 5C11 may encode noncoding RNA transcripts with a very restricted expression pattern**. (A) Longer exposure (10-day) of the Northern blot as shown in Figure 5B revealed the presence of short transcripts of 5C11. These heterogeneous short transcripts, marked by a bracket, were also down-regulated during ES cell differentiation by comparing Lanes 1, 2, 5 with Lanes 3, 4, 6. (B) Short transcripts of 5C11 were predicted to form very stable secondary structures. The nucleic acid folding program (mfold) was used for the prediction [47]. One predicted folded RNA structure for a full-length cDNA is shown here as an example. (C) A diagram is shown for the exon-intron organization of one of the isoforms of 5C11 transcripts. "Int" stands for the retroviral integration site in the ES clone 5C11. This isoform is predicted to form highly stable secondary structures (Figure 6B). (D) 5C11 transcripts are not detectable in any adult mouse organs by Northern blot. Total RNA samples were derived from the following adult mouse organs: Br, Brain; H, Heart; IN, Intestine; K, Kidney; Li, Liver; Lu, Lung; O, Ovary; S, Spleen; Te, Testis; Th, Thymus; U, Uterus. Total RNA was also derived from wild-type TC1 ES cells (TC1). The gel positions of the ribosomal RNA transcripts were marked.

Interestingly, transcripts derived from the novel gene trapped in the ES clone 5C11 appeared to be very heterogeneous with multiple transcription initiation sites, multiple polyA-addition signals and multiple alternative splicing patterns (Li and Leder, unpublished data). The main transcripts were apparently very large based on their positions relative to those of the ribosomal RNA transcripts (Figure 5B). However, a lot of the full-length cDNAs we isolated from the ES cells with 5' RACE and 3' RACE were around 500 bp in size. These cDNAs may correspond to the transcripts of lower molecular weights in the indicated region of Figure 6A when the same Northern blot shown in Figure 5C was subjected to longer exposure. These transcripts also appeared to be down-regulated during ES cell differentiation (Figure 6A). Intriguingly, these short full-length RNA molecules were predicted to form very stable secondary structures as exemplified by one of the RNA molecules (Figure 6B and Figure 6C). Consistent with its expression in the undifferentiated ES cells, several EST clones corresponding to this locus were derived from early blastocysts. However, no transcripts were detected by Northern blot in any major adult mouse organ (Figure 6D). Thus, this novel gene appears to be primarily expressed in the undifferentiated ES cells and blastocysts.

## Discussion

As has previously been shown, it is certainly possible to use a genomic approach to identify ES cell-specific genes at a much larger scale [33,34]. DNA micro-array as well as serial analysis of gene expression (SAGE) has been widely used in a variety of model systems. However, the retroviral gene trap approach has the advantage of being mutagenic, allowing us to directly evaluate the function of the genes *in vivo*. In addition, as demonstrated here and in other studies [20,35], this strategy can identify both novel genes as well as novel isoforms of known genes. For example, we have identified a novel embryonic isoform of *CSL/RBP-JKappa*. By contrast, DNA micro-array is based on known genes and available EST databases. SAGE analysis can generate previously unknown tags, but it is based on a short stretch of nucleotides at the 3'-end of a transcript and sometimes it is difficult to unequivocally locate the corresponding genes by simply searching the genome databases. The ability to identify novel tags in the SAGE analysis is limited by the abundance of the transcripts of a given gene. In the case of the novel embryonic isoform of *CSL/RBP-Jkappa *pulled out from our screen, it is unlikely that it would be discovered from the SAGE analysis of ES cells since it differs from other isoforms only at the extreme 5' exon. The DNA micro-array approach may also fail to identify this novel isoform unless oligonucleotides corresponding to this extreme 5' exon were included in the array.

The gene trap approach has its limitations. First, it is not designed as a large-scale approach as is DNA micro-array or SAGE and is limited by the efficiency of the vector to trap the endogenous genes. Second, although retroviral integrations are generally thought to be random, it appears that there are some hotspots for murine Moloney leukemia virus (MMLV) and other retroviruses [36]. It is possible that these drawbacks could be partially overcome by screening more ES clones.

The infection efficiency that was achieved with our current vector was still quite low based on the number of G418-resistant colonies obtained from a 10-cm dish of ES cells infected with this vector. It is true that ES cells are notoriously difficult to infect and normally their infection efficiencies are two to three orders of magnitude lower than those of fibroblast cells [37]. It is possible that we could increase the infection efficiencies by using VSVG pseudotyping which has been shown to be very effective in lentiviral vector-based infection of ES cells [38].

We favor our current reporter system expressing GFP and Neo as two independent proteins over a bi-functional reporter system involving the fusion of GFP and Neo reporter genes because we found that expression of the fused GFP and Neo reporter gene that we constructed is toxic to the cells even without G-418 drug selection (Supplemental Figure S3, see Additional file 1). Consistent with this, it was reported that a fusion protein consisting of GFP and Neo reporter genes displayed no visible GFP fluorescence although it had sufficient Neo activity [39].

We noticed that roughly one-fifth of the ES clones that had significant reduction of GFP fluorescence in FACS profiling were verifiable by Northern blot. One plausible reason for this is that FACS measures GFP fluorescence at the single cell level whereas Northern blot is based on the approximation of loading equal amount of total RNA derived from a group of cells. In this case undifferentiated ES cells proliferate very rapidly and it is likely that more ribosomal and other RNA transcripts involved in protein synthesis are produced in a given undifferentiated ES cell than are produced in a differentiated cell. Therefore, we may have under-estimated the amount of GFP reporter transcripts in the undifferentiated cells on a per cell basis by Northern blot. Another possible explanation is that the degree of reduction in GFP fluorescence varied greatly among the candidate ES clones. Indeed, almost all the clones that were verifiable by Northern blot were the ones that displayed dramatic down-regulation in FACS profiling when ES cells became differentiated.

It is also possible to carry out a similar screening in ES cells by using the well-established promoter-trap vectors containing a beta-geo marker gene encoding a β-galactosidase and Neo fusion protein, in combination with a fluorogenic β-galactosidase substrate [13,20,21,35,40]. However, it is much easier to detect GFP fluorescence by FACS and GFP fluorescence intensity is directly correlated with the amount of GFP protein produced in a given cell. In addition, the gene for β-galactosidase is much bigger than that of GFP. This may cause a big drop in the viral titer, hence the infection efficiency, due to the increased size of the corresponding insert. On the other hand, infection efficiency is important for retrovirus-based screening in ES cells for the reasons discussed above. Therefore, we prefer our current reporter system for gene trap-based screening in ES cells.

The novel embryonic isoform of *CSL/RBP-Jkappa *is very specific to undifferentiated ES cells. It is almost absent in differentiated cells. Other isoforms of *CSL/RBP-Jkappa *are generally thought to be ubiquitously expressed except that *RBP-2N *was highly enriched in the pre-B cell line 38B9 [41]. Interestingly, it was reported before that two monoclonal antibodies against *CSL/RBP-Jkappa *only stained the undifferentiated ES cells but not differentiated cells [42]. It remains to be determined whether the protein that these two monoclonal antibodies recognize corresponds to another isoform (or the same isoform identified in this study) of *CSL/RBP-Jkappa *expressed only in the undifferentiated ES cells. It is also intriguing that *CSL/RBP-Jkappa *and *LIN-12/Notch *signaling are involved in the generation and maintenance of definitive neural stem cells [43,44]. It is possible that this novel embryonic isoform of *CSL/RBP-Jkappa *may play a similar role in generation or maintenance of certain stem cells during embryonic development.

## Conclusion

ES cells share many characteristics with other somatic stem cells. They all have self-renewal and multipotential capacities. Many aspects of stem cell biology using ES cells will probably be applicable to other stem cells as well. However, unlike some other stem cells, ES cells can be readily maintained in culture and are amenable to genetic and molecular manipulations. The ability to generate germline transmissible mice from ES cells also gives us the unique opportunity to look into the phenotypes *in vivo*. The usefulness of this gene trap vector is manifested by its application in targeting the functional endogenous locus as well as introducing GFP and Neo reporters at the same locus (Li and Leder, unpublished data). The presence of the GFP reporter allows us to analyze the expression patterns *in vivo *and isolate the particular subset of cells expressing the candidate genes by using GFP-based cell sorting. Although we focused on ES cell-specific genes that are down-regulated during differentiation in this study, this gene trap system can also be used to identify genes that are up-regulated during ES cell differentiation (Li and Leder, unpublished data).

ES cells can be induced to differentiate into many cell lineages [45]. It is possible that some of the ES cell-specific genes identified in this study may have persistent expression in lineages which were not contained in the differentiation outcomes observed in this study. In this sense, ES cell differentiation can mimic many developmental processes *in vivo*. It is important to know how the undifferentiated state is maintained in ES cells and what initiates the decision to differentiate. A good starting point to answering these questions is to systematically survey ES cell-specific genes for their roles in maintaining the undifferentiated state as well as in promoting the differentiation of ES cells.

## Methods

### ES cell culture

Murine TC1 ES cells were grown on mitomycin C-treated feeder fibroblast cells. The medium used for undifferentiated ES cells is DMEM (Gibco) supplemented with 15% fetal bovine serum (Sigma) and 10^3^units/ml Leukemia Inhibitory Factor (LIF) (Chemicon). To obtain differentiated cells, the same growth medium was used and ES cells were grown without LIF and feeder fibroblast cells for four days.

### Construction of the gene trap vector

Inverse PCR was used to delete the intervening sequence in the original RET vector [23], resulting in the fusion of GFP and Neo reporter genes (Figure 1). The IRES sequence derived from EMCV was inserted at the junction in front of the start codon of Neo. The IRES sequence of the cellular *NF-kappaB *repressing factor (*NRF*) gene [46] was first amplified from a cDNA library by PCR and then placed in-between the splice acceptor site and GFP reporter.

### Retroviral infection and drug selection of ES cells

The gene trap vector was transfected into phoenix viral packaging cells [24] by using the calcium phosphate precipitation method. After culturing for 48 hours at 37°C, the viral supernatant was harvested and filtrated through 0.45 μm filter to remove cell debris. This viral filtrate was applied directly onto adherent ES cells grown in the presence of feeder fibroblast cells. G-418 was added to the medium 24 hours later after viral infection at the final concentration of 260 μg/ml. ES cells were subjected to the drug selection for 7 to 10 days until colonies appeared with a diameter of about 1 mm.

### FACS analysis of ES clones

G-418-resistant colonies were individually picked, partially digested with trypsin and transferred to 24-well plates seeded with feeder fibroblast cells. After growing for 3 to 5 days, half of the ES cells derived from each clone were frozen as a stock and the rest were split into two wells of 24-well plates. One portion of the cells was grown in the medium with feeder fibroblast cells plus LIF and the other was grown without feeder cells and LIF. After being cultured for 4 days, ES cells from both undifferentiated and differentiated populations were harvested for FACS analysis.

### RNA preparation and Northern blot

Trizol reagent (Invitrogen) was used to harvest total RNA from ES cells. After purification, total RNA was dissolved in DEPC-treated water. The concentration of RNA was measured with spectrophotometer and approximately equal amounts of total RNA for differentiated and undifferentiated samples were loaded for Northern blot analysis.

Total RNA was also prepared from the adult mouse organs with Trizol reagent.

### Amplification of the integration sites and flanking genomic sequences by inverse PCR

Genomic DNA was prepared from each candidate ES clone and subjected to HindIII restriction enzyme digestion. After HindIII was heat-inactivated, the digested genomic DNA was treated with T4 DNA ligase. The ligation mixture was used directly as the DNA template for the inverse PCR reactions. Two rounds of the inverse PCR reactions were performed, with the PCR reaction mixture from the first round inverse PCR as the template for the second round inverse PCR by using two nested primers. The two primers used for the first round inverse PCR reaction are IR(N)-GF1 and U5-R1, with the sequences of 5'-cctgccacagacttagaatcagcc and 5'-ctcttgcagttgcatccgacttgtg, respectively. The two primers used for the nested second round inverse PCR are IR(N)-GF2 and U5-R2, with the sequences of 5'-ctctaaggaccctgattcc and 5'-gtggtctcgctgttccttg, respectively.

### RT-PCR analysis of the fusion transcripts

The primer used for the first-strand cDNA synthesis is Xho-Junc with the sequence of 5'-tcgagccctgagccgta which is complementary to the sequence at the junction of SA and I(N) in the gene trap vector eGeoN/E+pA. The reverse primer used for the PCR reaction is Bcl-2R2 with the sequence of 5'-ccgtacagttccacaaag which is complementary to the sequence present in the exon 3 of *bcl-2 *in the SA region of the gene trap vector. The forward primers used for PCR are gene-specific primers derived from sequences present in the exons of the predicted endogenous genes upstream of the gene trap integration sites in these ES clones. For the ES clone 2G2 and 5C25, it is P1 with the sequence of 5'-tgagaagcccaggcttctctg. For the ES clone 5C1 and 5C11, they are 5C1-F1 of 5'-agtctcttgccttctcagg and 5C11-F1 of 5'-cctttttggaatggagacagcag, respectively.

### Semi-quantitative RT-PCR analysis of the different isoforms of *CSL/RBP-Jkappa*

First-strand cDNA template was synthesized from 2 μg total RNA of undifferentiated wild-type TC1 ES cells by using Superscript II™ (Invitrogen/Gibco). One-tenth of the first-strand cDNA mixture was used in the RT-PCR reactions. Thirty cycles of amplification were performed with the Advantage GC cDNA polymerase (BD Biosciences Clontech). The primers used are P1 5'-ccttcaagatatccagcaag, P2 5'- cctataggccaacatttgag, P3 5'- caaacgactcactagggaag, P4 5'- cacagggttgagactcttg and P5 5'- gtaatgccctccggttttcctc.

## Authors' contributions

XL conceived of the study and PL participated in its design. XL carried out all the experimental studies. Both XL and PL read and approved the final manuscript.

## Supplementary Material

Additional file 1 Supplemental figures. Northern blot as shown in Supplemental Figure S1 was used to examine the levels of GFP hybrid transcripts of some candidate ES clones that display significant reduction of GFP fluorescence in FACS profiling. Supplemental Figure S2 shows the GFP FACS profiles of both undifferentiated cells and differentiated cells derived from the candidate ES clone 5C32. Supplemental Figure S3 provides evidence suggesting that an EGFP/Neo fusion protein is toxic to the cells. Click here for file
