# Supplementary material for: Identifying genes preferentially expressed in undifferentiated embryonic stem cells
Source: BMC Cell Biol. 2007 Aug 28;8:37. doi: 10.1186/1471-2121-8-37 (PMC1995199; doi:10.1186/1471-2121-8-37)
Supplement: Additional file 1 — Supplemental figures. Northern blot as shown in Supplemental Figure S1 was used to examine the levels of GFP hybrid transcripts of some candidate ES clones that display significant reduction of GFP fluorescence in FACS profiling. Supplemental Figure S2 shows the GFP FACS profiles of both undifferentiated cells and differentiated cells derived from the candidate ES clone 5C32. Supplemental Figure S3 provides evidence suggesting that an EGFP/Neo fusion protein is toxic to the cells. [file 1471-2121-8-37-S1.pdf]

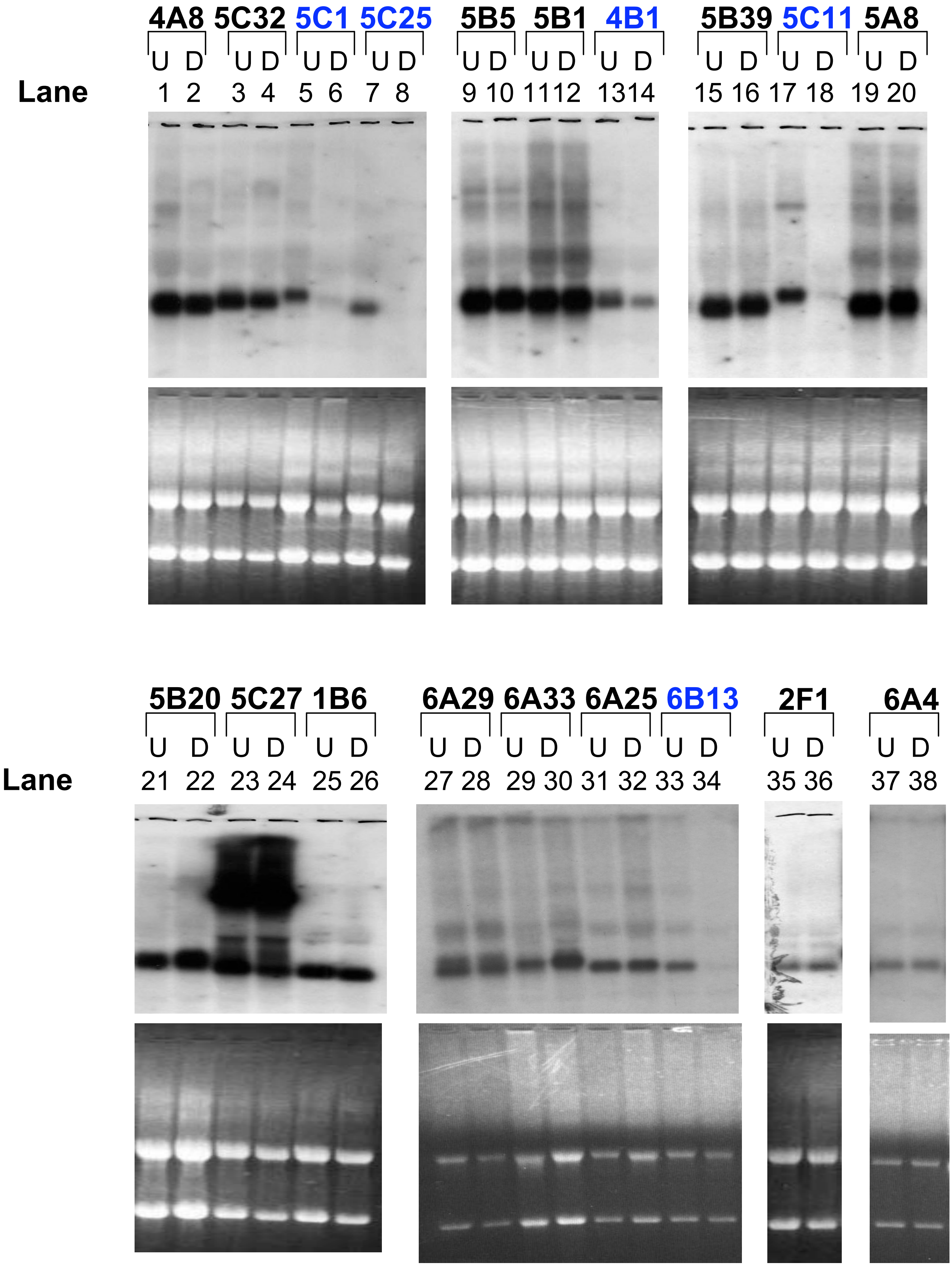

Supplemental Figure S1

A

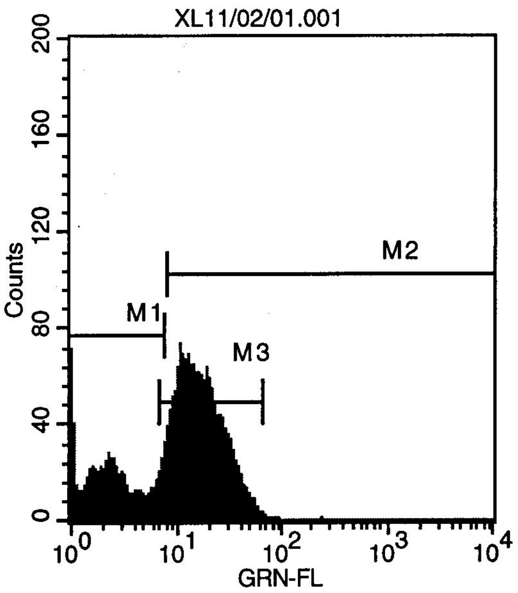

Gate: G1	Gated Events: 10000
Total Events: 18781	

Marker	Left, Right	Events	% Gated	% Total	Mean
All	1, 9910	10000	100.00	53.25	14.62
M1	1, 8	2606	26.06	13.88	3.24
M2	8, 9910	7274	72.74	38.73	18.81
M3	7, 66	7641	76.41	40.68	18.17

5C32 (undifferentiated)

B

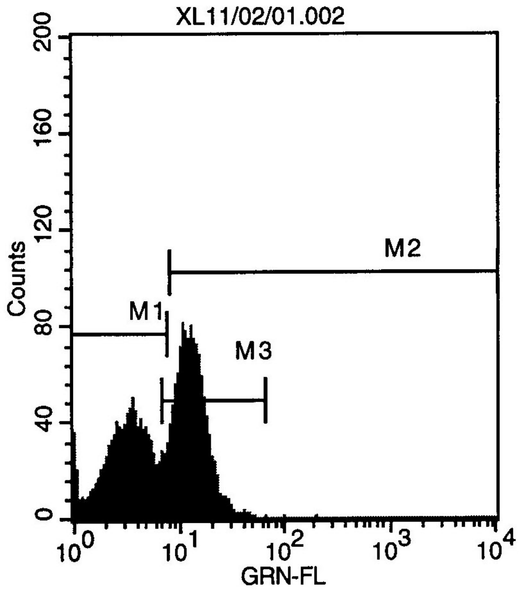

Gate: G1	Gated Events: 10000
Total Events: 26829	

Marker	Left, Right	Events	% Gated	% Total	Mean
All	1, 9910	10000	100.00	37.27	9.10
M1	1, 8	4510	45.10	16.81	3.67
M2	8, 9910	5372	53.72	20.02	13.68
M3	7, 66	5751	57.51	21.44	13.23

5C32 (Differentiated)

Supplemental Figure S2

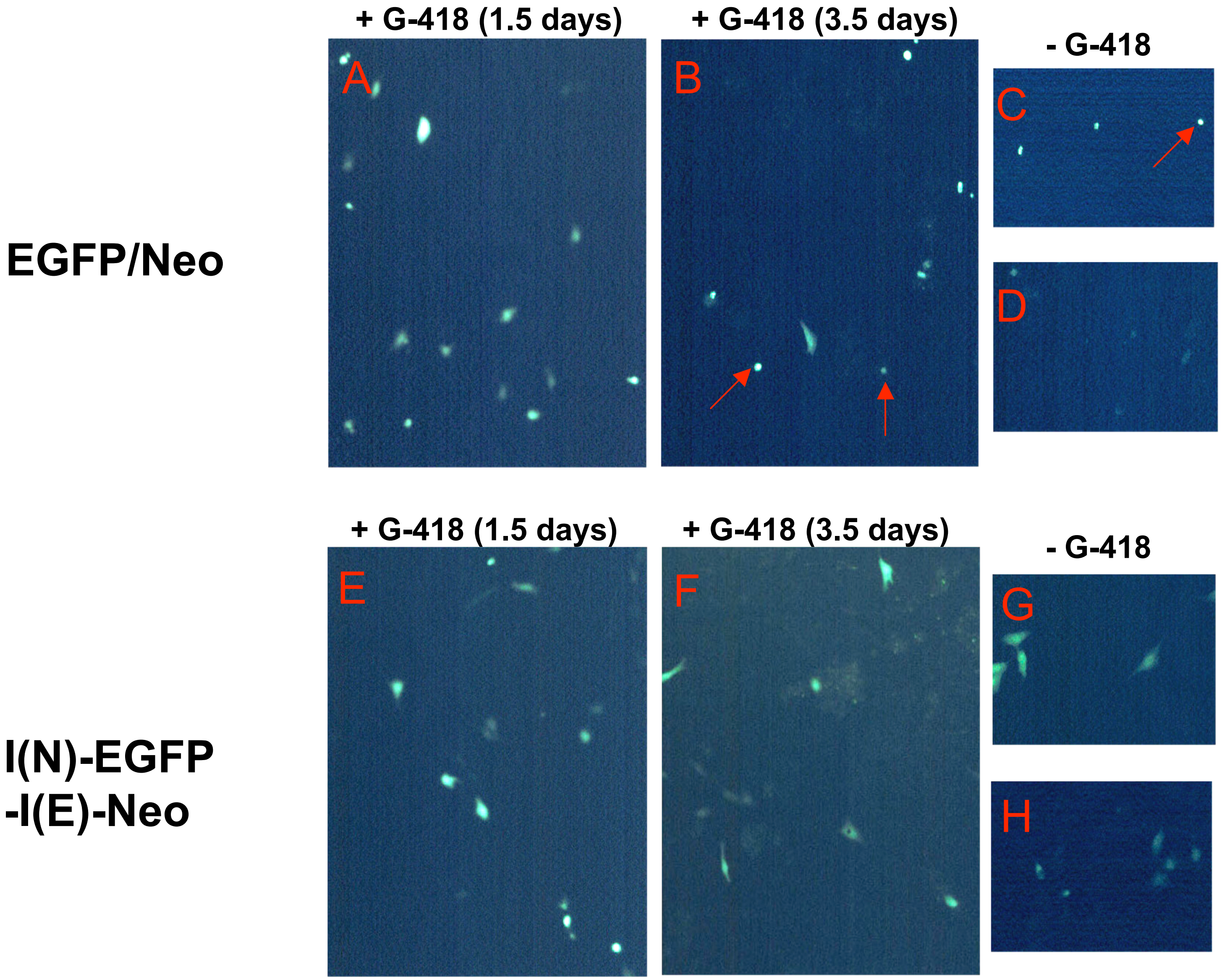

Supplemental Figure S3

Supplemental Figure legends

Supplemental Figure S1. Northern blot analysis of the candidate ES clones with a GFP cDNA fragment as the probe. U, Undifferentiated ES cell population. D, Differentiated ES cell population. Approximately 10 μg of total RNA was loaded for the U and D samples of each ES clone. Except for the ES clone 1B6 and 6A29, all other 19 ES clones shown on this Northern blot displayed significant reduction of GFP fluorescence by FACS when differentiated. ES clones whose GFP hybrid transcript was confirmed to be down-regulated during differentiation by Northern blotting are highlighted.

Supplemental Figure S2. GFP FACS profiling of the ES clone 5C32. The horizontal axis indicates the intensity of GFP fluorescence (GRN—FL) whereas the vertical axis stands for the cell number (Counts). M1, GFP-negative cells. M2, GFP-positive cells. M3 marks the boundaries of the peak that contain the GFP-positive cells.

A, a FACS profile of the undifferentiated ES cells for the ES clone 5C32 which were grown in the presence of LIF and feeder fibroblast cells.

B, a FACS profile of the differentiated cells for the ES clone 5C32 which were grown without LIF and feeder fibroblast cells.

Supplemental Figure S3. Over-expression of an EGFP/Neo fusion protein is toxic to the cells.
3T3 fibroblast cells were transfected with an expression vector either constitutively expressing an EGFP/Neo fusion protein or expressing EGFP and Neo as two independent protein product from the I(N)-EGFP-I(E)-Neo cassette used in this study (see Figure 1). Green fluorescent cells shown in the panels A, B, C and D express the EGFP/Neo fusion protein and those in the panels E, F, G and H express GFP from the I(N)-EGFP-I(E)-Neo cassette. Cells shown in the panel D and H are stably selected G-418-resistant clones. Green fluorescent cells in the other panels (A, B, C, E, F, G) are transiently transfected 3T3 fibroblast cells. Cells in A and E were subjected to G-418 selection for one and half days while those in B and F were selected with G-418 for three and half days. Cells in C and G were without G-418 selection. Many dying fluorescent cells (red arrow) were observed when the EGFP/Neo fusion protein was over-expressed.
